# An improved, low-cost, hydroponic system for growing Arabidopsis and other plant species under aseptic conditions

**DOI:** 10.1186/1471-2229-14-69

**Published:** 2014-03-21

**Authors:** Fulgencio Alatorre-Cobos, Carlos Calderón-Vázquez, Enrique Ibarra-Laclette, Lenin Yong-Villalobos, Claudia-Anahí Pérez-Torres, Araceli Oropeza-Aburto, Alfonso Méndez-Bravo, Sandra-Isabel González-Morales, Dolores Gutiérrez-Alanís, Alejandra Chacón-López, Betsy-Anaid Peña-Ocaña, Luis Herrera-Estrella

**Affiliations:** 1 Laboratorio Nacional de Genómica para la Biodiversidad (Langebio)/Unidad de Genómica Avanzada (UGA), Centro de Investigación y Estudios Avanzados del IPN, 36500 Irapuato, Guanajuato, México; 2 Current address: Department of Biological and Environmental Sciences, Institute of Biotechnology, University of Helsinki, 00014 Helsinki, Finland; 3 Current address: Instituto Politécnico Nacional, Centro Interdisciplinario de Investigación para el Desarrollo Integral Regional Unidad Sinaloa, 81101 Guasave, Sinaloa, México; 4 Current address: Red de Estudios Moleculares Avanzados, Instituto de Ecología A.C. Carretera Antigua a Coatepec #351, Xalapa 91070, Veracruz, México; 5 Current address: Instituto Tecnológico de Tepic, Laboratorio de Investigación Integral en Alimentos, División de Estudios de Posgrado, 63175 Tepic, Nayarit, México

**Keywords:** Hydroponics, Arabidopsis, Root, Phosphate starvation, Pathogenesis

## Abstract

**Background:**

Hydroponics is a plant growth system that provides a more precise control of growth media composition. Several hydroponic systems have been reported for Arabidopsis and other model plants. The ease of system set up, cost of the growth system and flexibility to characterize and harvest plant material are features continually improved in new hydroponic system reported.

**Results:**

We developed a hydroponic culture system for Arabidopsis and other model plants. This low cost, proficient, and novel system is based on recyclable and sterilizable plastic containers, which are readily available from local suppliers. Our system allows a large-scale manipulation of seedlings. It adapts to different growing treatments and has an extended growth window until adult plants are established. The novel seed-holder also facilitates the transfer and harvest of seedlings. Here we report the use of our hydroponic system to analyze transcriptomic responses of Arabidopsis to nutriment availability and plant/pathogen interactions.

**Conclusions:**

The efficiency and functionality of our proposed hydroponic system is demonstrated in nutrient deficiency and pathogenesis experiments. Hydroponically grown Arabidopsis seedlings under long-time inorganic phosphate (Pi) deficiency showed typical changes in root architecture and high expression of marker genes involved in signaling and Pi recycling. Genome-wide transcriptional analysis of gene expression of Arabidopsis roots depleted of Pi by short time periods indicates that genes related to general stress are up-regulated before those specific to Pi signaling and metabolism. Our hydroponic system also proved useful for conducting pathogenesis essays, revealing early transcriptional activation of pathogenesis-related genes.

## Background

Standardization of growth conditions is an essential factor to obtain high reproducibility and significance in experimental plant biology. While lighting, humidity, and temperature are factors that can be effectively controlled by using plant growth chambers or rooms, media composition can be significantly altered by the physiochemical characteristics and elemental contaminants of different batches of gelling agents [1,2].

For example, the inventory of changes in root system architecture (RSA) as a plant adaptation to nutrient stress can be influenced by the presence of traces of nutrients in different brands or even batches of agar as reported for the Pi starvation response [1]. Detailed protocols for obtaining real nutrient-deficient solid media for several macro and micronutrients have been recently reported [1,2]. These protocols describe a careful selection of gelling agents based on a previous chemical characterization that increase the cost and time to set up experiments. In addition those problems associated with media composition, plant growth window is reduced in petri plates (maximum 2–3 weeks) [3]. In vitro culture time can be extended using glass jars but accessibility to the root system is then compromised. Furthermore, additional handling and thus unnecessary plant stress during seedlings transfer to new growth media as well as during plant material collection should be also considered when experiments on solid media are designed.

One strategy for circumventing all problems described above is the use of hydroponic systems for plant culture. Several hydroponic systems have been reported for Arabidopsis [4-13] and some of them are now commercially available (Aeroponics®) [12]. Most of these systems are integrated by a plastic, glass or polycarbonate container with a seed-holder constituted by rock wool, a polyurethane (sponge) piece, a steel or nylon mesh, polyethylene granulate, or a polyvinyl chloride (PVC) piece. Those are open systems, which allow axenic conditions or reduced algal contamination into liquid growth media but sterility is not possible.

Here, we describe step by step a protocol for setting up a simple and low-cost, hydroponic system that allows sterility conditions for growing Arabidopsis and other model plants. This new system is ideal for large-scale manipulation of seedlings and even for fully developed plants. Our system is an improved version of Schlesier *et al*. [8], in which the original glass jar and steel seed-holder are substituted by a translucent polypropylene (PE) container and a piece of high-density polyethylene (HDPE) mesh. All components are autoclavable, reusable, cheap, and readily available from local suppliers. The new device designed as seed-holder avoids the use of low-melting agarose as support for seeds, allowing a quick and easy transfer to new media conditions and/or harvest of plant material. The efficiency and functionality of our proposed system is demonstrated and exemplified in experiments that showed typical early transcriptional changes under Pi starvation and pathogen infection.

## Results and discussion

### Description of the hydroponic system

**Figure 1:**
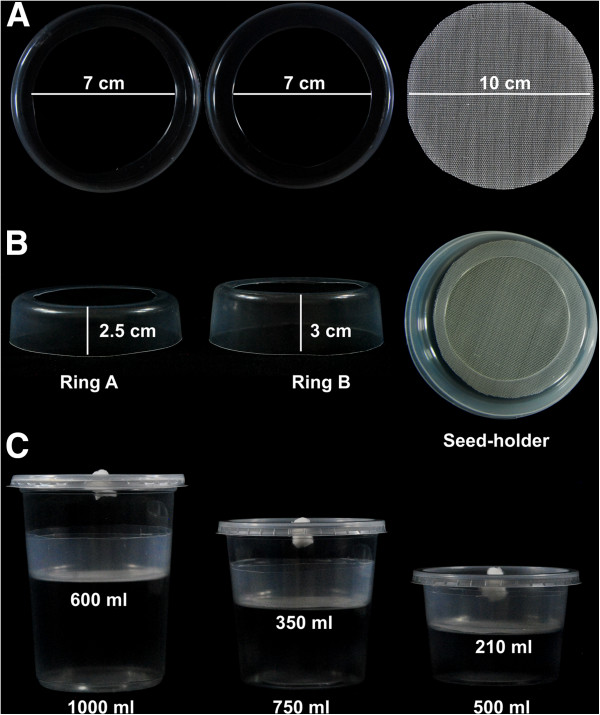
**Hydroponic system: component dimensions and assembly. A and B)** Dimensions and assembly of seed-holder. **C)** Assembled hydroponic system. Containers with different volume for liquid media are shown. The numbers at the bottom’s container indicate the maximum volume and the number inside the container the volume of liquid media used in each case.

We have improved a previously reported hydroponic system, consisting of a glass jar and stainless piece integrated by a wire screen fixed between two flat rings and held in place by three legs [8], by a simpler and cheaper system assembled with a PE vessel and a seed-holder integrated by a circle-shape HDPE mesh and two PE rings (Figure 1A,B; Table 1). Vessels and mesh used here are readily available in local markets; vessels are actually food containers (Microgourmet®, Solo Cup, USA, http://www.solocup.com) available in food package stores while the HDPE mesh is a piece of anti-aphid mesh acquired in local stores providing greenhouse supplies (http://www.textilesagricolas.mx). A small cotton plug-filled orifice in the container lid allows gas exchange to the system (Figure 1C). This ventilation filter reduces but does not eliminate high humidity in the medium container. Such problem could be solved adding more ventilation filters or using other sealing materials as micropore 3 M® paper tape. Aeration of the liquid medium is not required for our hydroponic system. No negative effects on plant growth have been observed when small tanks are used as medium containers (references in Table 1).

**Table 1:** Comparison between hydroponic systems previously reported and the system proposed here

**Parameter**	**Agar-filled plastic holder**	**Rockwool-filled plastic holder**	**Sponge into a polypropylene sheet**	**Polyethylene granulate**	**Stainless mesh fixed two metal rigs/Nylon mesh on photo slide mount**	**This system**
**Liquid medium container**	Plastic box	Plastic box	Magenta GA-7 vessel®	Glass vessel	Round-rim glass jars/glass vessel	Plastic container
**Costs**	Intermediate to high	Intermediate	High	High	High	Low
**Setup time**	Intermediate to high	Intermediate	Low	Low	High	Low to intermediate
**Reuse of seed-holder**	No	No	No	No	Yes/No	Yes
**Throughput**	Intermediate	Intermediate	High	High	High/intermediate	Intermediate
**Container volume**	Small to high	Small to intermediate	Small	Small to high	Intermediate	Intermediate to high
**Medium evaporation**	High	High	Low	High	Low/High	Low
**Seedling number per holder**	One	One	One	Many	Many	Many
**Sterility**	No	No	Yes	No	Yes/No	Yes
**Aeration**	Yes/No	Yes/No	No	No	No	No
**Time for moving and sampling large batches of plants between media**	High	High	High	High	High	Low
**Development window**	Adult plants	Adult plants	Seedling to adult plants	Seedling	Seedling	Seedling to adult plants
**References**	[3,9,12]	[4,10,11]	[6]	[7]	[5,8,13]	

**Figure 2:**
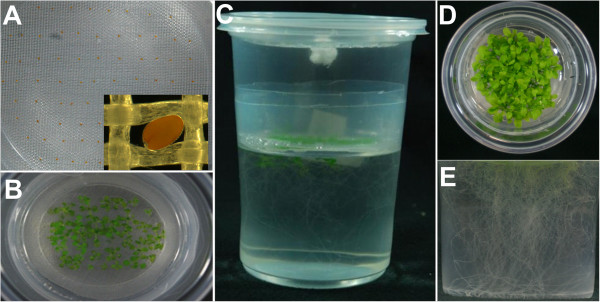
**Arabidopsis seedlings growing under the hydroponic system proposed. A)** Seeds sown on the mesh’s seed-holder. A close-up view of a single seed is shown (inset). **B-E)** Seedlings growing in our hydroponic system. Top **(B)** and lateral view **(C)** of 12-day-old seedlings. Top **(D)** and lateral view **(E)** of 3-weeks-old seedlings.

The new seed-holder for positioning seeds on top of the liquid medium consists of a mesh of HDPE monofilaments held between two PE rings (ring A and B), with an area of 78.54 cm^2^ (diameter =10 cm) which is able to hold 50 to 65 Arabidopsis seedlings for up to 10–15 days after germination (Figure 1A,B; Figure 2) (Table 1). Fully developed Arabidopsis plants (2–3 plants per vessel) can also be grown in this system if the container lid is replaced by another PE container (Additional file 1). Anti-aphid mesh with a 0.75 mm by 0.75 mm opening size (mesh usually named 25 × 25) is adequate for keeping Arabidopsis seeds on top of the mesh (Figure 2A,B) and allowing independent root system development (Figure 2C,D,E). Anti-aphid or anti-insect mesh with lower density can be useful for seeds larger than Arabidopsis seeds. No legs for supporting the mesh-holder are needed in our hydroponic system. The seed-holder is just placed into the container and kept in place by pressing against the container walls. Unlike other protocols previously reported (Table 1), the container size of the system described here can vary according to volume of medium required (Figure 1C). However, the same standard seed-holder can be used for 1000 ml, 750 ml, or 500 ml containers, giving an effective volume for root growth of 600 ml, 350 ml and 210 ml, respectively (Figure 1C).

**Figure 3:**
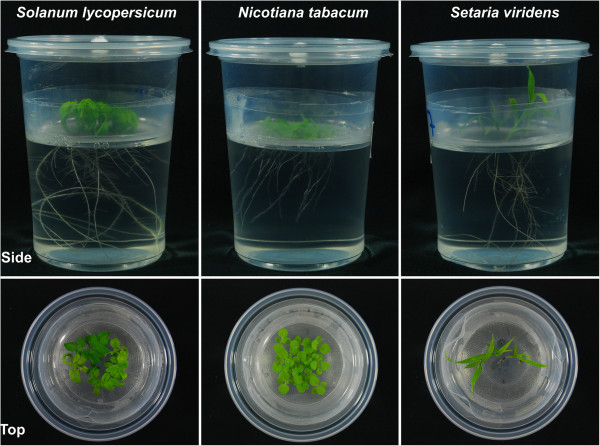
**The hydroponic system proposed can be used with other model monocot and dicot plants.** Lateral and top views of root and shoot growth of *S. lycopersicum*, *N. tabacum*, and *S. viridis* at 2 to 3 weeks old.

Our hydroponic system can be used for growing other model species under aseptic conditions. *Solanum lycopersicum*, *Nicotiana tabacum,* and *Setaria viridis* seeds were sterilized and directly sowed on the mesh. For all species, an adequate growth of shoot and root system was observed two weeks after germination (Figure 3).

**Figure 4:**
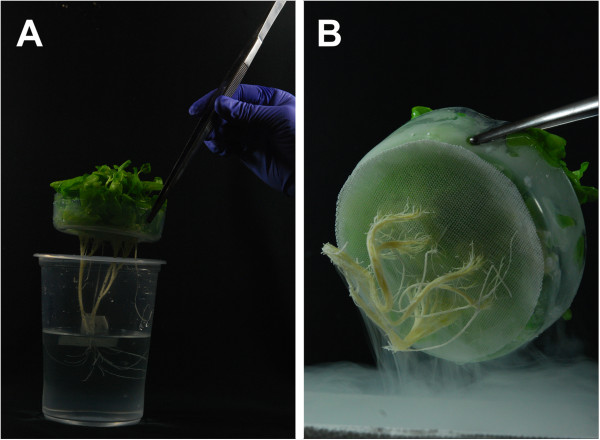
**An easy and quick transfer to new growth media and/or root harvesting can be carried out with this hydroponics system. A)** Tobacco seedlings are transferred handling the seed-holder only. **B)** Batch of tobacco seedlings growing on the seed-holder frozen into liquid nitrogen.

Other advantages of this hydroponic system are related to plant transfer and plant tissue collection. For both, only a dressing tissue forceps (6 or 12 inch), previously sterilized, is required to pull up the seed-holder, and place it into new media (Figure 4A) or to submerge it into a liquid nitrogen container for tissue harvest (Figure 4B). Root harvest of young seedlings of the hydroponic system is also easier and less time-consuming than those from seedlings grown in agar media. When the seed-holder is taken out from the container, young roots adhere to mesh and can be blotted with an absorbent paper towel and immediately frozen in liquid nitrogen. Shoot biomass can be also easily detached from the mesh using a scalpel and then the mesh with the attached roots can be processed separately.

### Protocol for setting up the hydroponic system

Step by step instructions for set up of hydroponic system are indicated in the following section and the Additional file 2. Tips and important notes are also indicated.

1. Getting a nylon mesh (See Figure 1A)

Get a piece of anti-aphid or anti-insect mesh. Draw a circle (10 cm diameter) using a marker and a cardboard template. Trim the circle using a fork. After tripping the circle, remove color traces on mesh using absolute alcohol. Wash the mesh under running water (Option: Use deionized water). Dry on paper towels. Tip: Use a red color marker for drawing. Red color is easier to clean than other colors.

2. Making a mesh holder (See Figure 1A,B)

Cut the 500 ml PE container's bottom. Use a scalpel blade. Leave a small edge (0.5 cm width). The mesh circle will put on this edge. For ring A, leave a height of 2.5 cm, for ring B leave 3 cm. Tip: Use a scalpel blade with straight tip to cut easily the container's bottom.

3. Preparing the container lid

Locate the center of container lid and mark it. Drill the lid center. Seal the small lid hole with a cotton plug. Tip: Use a hot nail to melt a hole in the lid to avoid burrs.

4. Sterilization

Container and rings and mesh have to be separately sterilized by autoclaving (121°C and 15 psi pressure by 20 minutes). Put container, ring, and mesh groups into poly-bags. For container and rings, close but not seal the poly-bags. If so, pressure variations during sterilization could damage them. Important point: Put the autoclave in liquid media mode. Tip: After sterilization, put poly-bags into another bag for reducing contamination risks.

5. Hydroponic system assembly (See Figure 1C)

Open the sterilized poly-bags containing containers, rings, meshes, and lids. Put a volume of previously sterilized liquid medium into the container. Tip: the use liquid media at room temperature reduces the steam condensate on container lid and walls. Take a ring B with a dressing tissue forceps and put it into the container just above the liquid media level. Put a mesh piece on the ring B, lift it slowly and then return it on the ring avoiding to form bubbles. Fit the ring A onto the mesh piece. Tip: If it is difficult to fit the ring A onto the mesh piece, warm the ring quickly using a Bunsen burner. Finally, close the container.

### Applications of our hydroponic system: 1) Quick transcriptional responses to Pi starvation

Applications of this new hydroponic culture system for model plants were analyzed in this study. Changes during Pi starvation at the transcriptional level associated with the Arabidopsis RSA modifications have been previously described [13]. Here, first we compared the effects of Pi-availability on RSA and the expression profiles of eight marker genes for Pi deficiency in Arabidopsis seedlings grown in hydroponics versus agar media. Then, taking advantage of the short time that is required with this new hydroponic system for transferring plants to different media, early transcriptional responses to Pi depletion were explored at the genome-wide level; such responses have not been previously evaluated.

#### *Arabidopsis growth and Pi-depletion responsive genes on Pi-starved hydroponic media*

**Figure 5:**
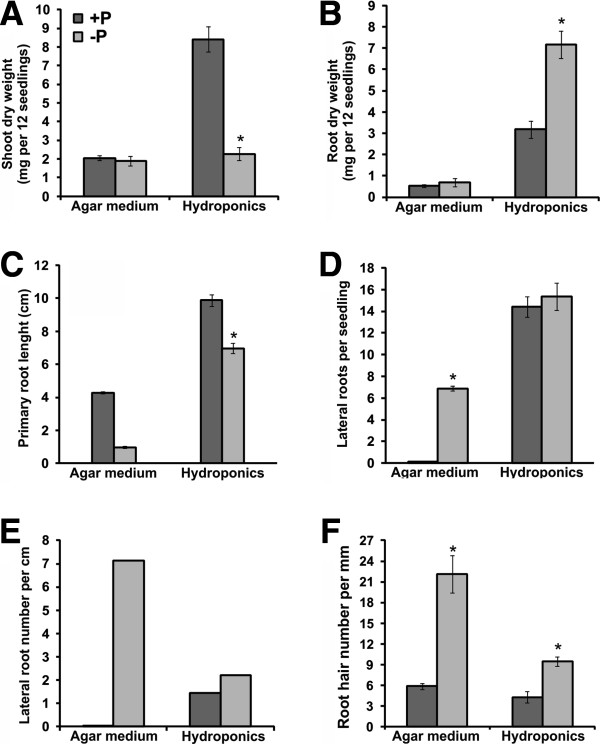
**Plant growth under hydroponics or solid media under contrasting Pi regimens (A-F).** Arabidopsis seedlings were directly sowed on the seed-holder (50 – 60 seed per mesh) or agar media (30–35 seeds per plate), growth for 12 days under two different Pi regimens (-P = 10 μM Pi, +P = 1.25 mM Pi) and then analyzed. Bars represent means ± SE (Hydroponics, biological replicates = 5, n = 20–60; agar medium, biological replicates = 10–15, n = 15). Asterisks denote a significant difference from corresponding control (+P treatment) according Student’s t test (P<0.05).

Arabidopsis seeds were germinated and grown for 12 days in hydroponics or agar media containing high-Pi (1.25 mM) or low-Pi (10 μM) concentrations as previously reported [14,15]. By day 12 after germination, a higher shoot and root biomass was produced by Arabidopsis seedlings grown in hydroponics than those grown in solid media (Figure 5A,B), which is consistent with previous comparisons between both methods for growing Arabidopsis [5]. The typical increase in root biomass accumulation under Pi stress was observed in seedlings grown in agar medium, however such change was not statistically significant (Figure 5B). In contrast, the dry weight of roots of seedlings grown in hydroponics under Pi stress was 2.25-fold higher compared to that observed for Pi-sufficient seedlings (Figure 5B). This higher root growth under low-Pi is a typical RSA change that allows an increase of Pi uptake under natural soil conditions [14]. Regarding RSA adaptation to low Pi availability, we also found a 30% reduction in primary root length with respect to control treatment under hydroponics while such reduction was higher (76%) in roots from agar media (Figure 5C). Similarly, there was a modest increase in lateral root and root hair density under low-Pi in liquid media whereas a marked increase under same Pi growth condition was found in agar media (Figure 5D,E,F).

Although the effects of Pi deficiency on root development were more severe in agar media than in our hydroponic system, the typical root modifications induced by Pi stress (primary root shortening and higher production of lateral roots and root hairs) [14], were observed in both systems. Differences in the magnitude of RSA alterations in response to Pi-deprivation could be explained by variations in medium composition caused by gelling agents added, and/or the ease to access to Pi available in the growth systems used. It has been previously shown than contaminants such as Pi, iron, and potassium in the gelling compounds can alter the morphophysiological and molecular response to Pi starvation [1]. Hydroponics provides a better control on media composition and allows a direct and homogenous contact of the whole root system with the liquid medium. This condition could be improve nutrient uptake, and under Pi starvation, alleviate the dramatic changes of RSA observed usually in roots of seedlings grown in agar media.

**Figure 6:**
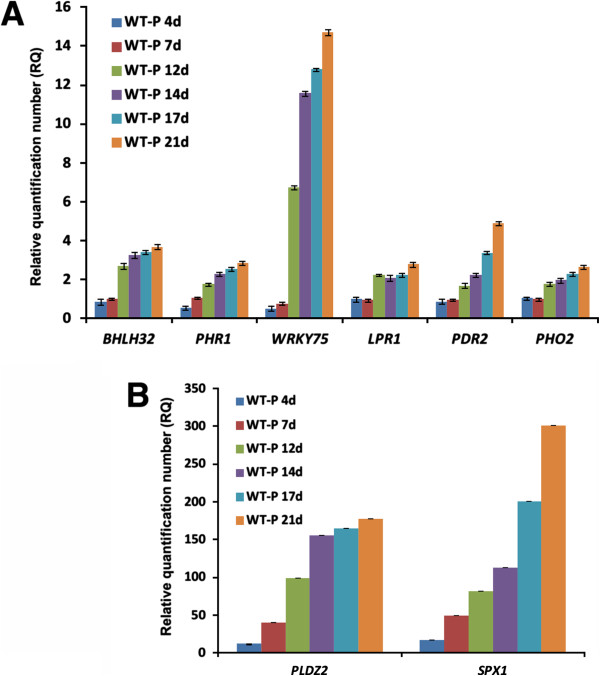
**qRT-PCR expression profiling of marker genes for Pi starvation in Arabidopsis seedlings grown hydroponically.** Expression profiling of **A)** transcriptional modulators and genes involved with root meristem growth and **B)** Pi signaling and recycling. Arabidopsis seedlings were grown for 21 days under two different Pi regimens (-P = 10 μM Pi, +P = 1.25 mM Pi). RNA of whole seedlings was extracted at six time points and gene expression levels were analyzed by qRT-PCR assays. Relative quantification number (RQ) was obtained from the equation (1 + E)2^∆∆CT^ where ∆∆CT represents ∆CT(-P)–∆CT(+P), and E is the PCR efficiency. C_T_ value was previously normalized using the expression levels of ACT2, PPR and UBHECT as internal reference. Data presented are means ± SE of three biological replicates (n = 100-150).

Afterwards, we determined the efficiency of the hydroponics system for inducing expression of low-Pi-responsive genes. Analysis of the expression profiles for eight genes involved with transcriptional, metabolic and morphological responses to Pi starvation were carried out in whole Arabidopsis seedlings that were grown in either low or high-Pi hydroponic conditions at 4, 7, 12, 14, 17 and 21 days. Transcript level quantification of the transcriptional factors (TF) *PHR1* (PHOSPHATE STARVATION RESPONSE 1), *WRKY75* (WRKY family TF) and *bHLH32* (basic helix-loop-helix domain-containing TF) revealed a direct influence of Pi stress persistence on the up-regulation of these three molecular modulators [16-18]. *WRKY75* had the highest expression level among the TFs analyzed with a significant induction in expression after 12 days under Pi deficiency (Figure 6A). *BHLH32* showed a similar increase in expression. As most molecular responses to Pi starvation are affected in *phr1* mutant, *PHR1* has been considered a master controller of Pi signaling pathway [16,19]. In the case of *PHR1* we found that this gene did not show as constitutive expression under Pi deficiency as originally reported [16]. Instead, the expression profile of this master regulator in roots showed responsiveness to low-Pi conditions (Figure 6A). These data are consistent with the low transcriptional induction of *PHR1* previously observed in Arabidopsis shoots [20]. *LPR1 (LOW PHOSPHATE 1)* and *PDR2 (PHOSPHATE DEFICIENCY RESPONSE 2)*, two genes involved in root meristem growth [21], and the E2 ubiquitin conjugase *PHOSPHATE 2* (*PHO2/UBC24)*, related with Pi loading [22], showed a notable increase in expression after 14 d of treatment (Figure 6A). In contrast, *SPX1 (a gene encoding a protein with a SYG1/Pho81/XPHR1 domain)* and *PLDZ2 (PHOSPHOLIPASE DZ2)*, two typical marker genes of Pi deficiency implicated with Pi signaling and recycling [23,24] respectively, showed a significant induction starting at day four. Both *SPX1* and *PLDZ2*, but especially *SPX1*, had a marked increase in expression level (Figure 6B). The expression analysis of these Pi-responsive genes together with RSA analyses during Pi starvation on hydroponics demonstrate the high performance of our system for plant growing and for analyzing molecular responses to nutrimental deficiency.

#### *Exploring early genome-wide transcriptional responses to Pi depletion: overview and functional classification of differentially expressed genes*

**Figure 7:**
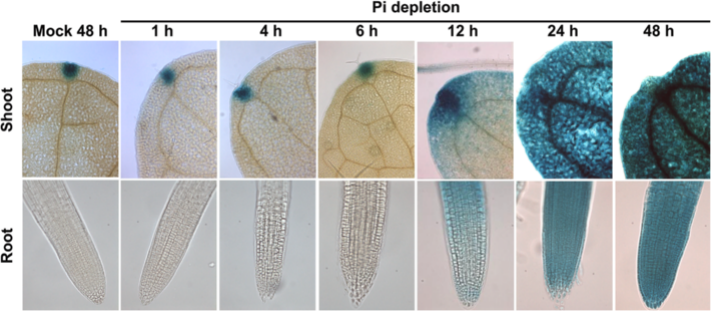
**AtPT2::GUS expression pattern under Pi depletion.** Arabidopsis AtPT2::GUS seedlings were grown hydroponically for 12 days under sufficient Pi level (125 μM Pi) and then transferred to Pi-depleted liquid media or control media (125 μM Pi, mock). GUS activity in leaves and root was monitored by histochemical analyses at different time points. GUS expression in the mock condition is shown for the last time sampled (48 h).

Early transcriptional responses to Pi availability at the genome-wide level (4 h to <12 h) have been previously determined in whole Arabidopsis seedlings using microarray platforms [25,26]. An important experimental condition in those studies has been the use of a 100–200 μM as a low-Pi concentration, considered enough to support biomass accumulation but not to induce an excessive Pi accumulation [26]. It has been reported that Arabidopsis seedlings growing at 100 μM Pi in agar media had similar endogenous phosphorus (P), biomass production and RSA to those growing at 1 mM Pi [14]. In liquid media, 200 μM Pi has also been considered as a Pi-sufficient condition for growing monocot species such as maize [27]. We found that Arabidopsis seedlings grown with150 μM Pi in liquid media are not able to induce the expression of *AtPT2*/AtPHT1;4 (*PHOSPHATE TRANSPORTER 2*), a high-affinity Pi transporter responsive to Pi starvation reviewed in [28] as revealed by analysis of Arabidopsis seedlings harboring the transcriptional AtPT2::GUS reporter. Seedlings growing in hydroponics during 12 days showed null expression of the reporter in either shoot or root. When these seedlings were transferred to Pi-depleted media, AtPT2::GUS reporter was detected 12 h after transfer (Figure 7).

**Figure 8:**
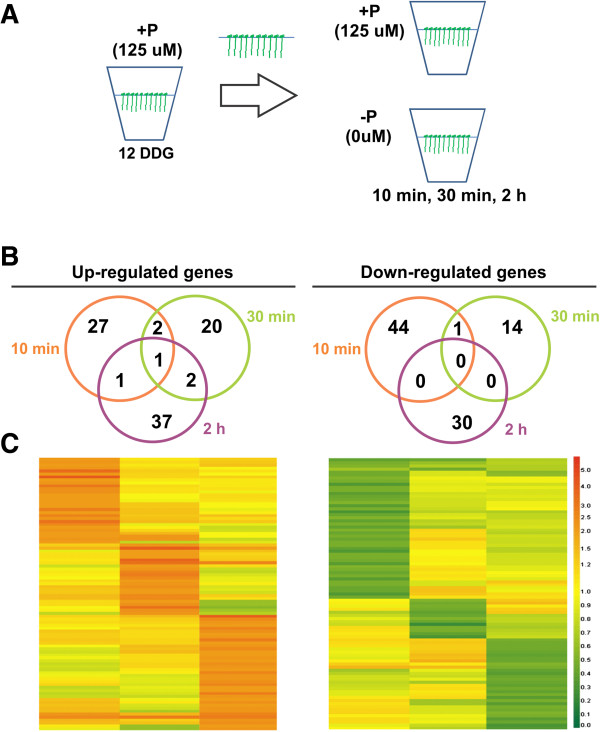
**Early changes in the transcriptome of Arabidopsis roots under Pi starvation. A)** Workflow for experiments. Arabidopsis seedlings were grown hydroponically for 12 days under sufficient Pi level (125 μM Pi) and then transferred to Pi-depleted liquid media for short times. Roots were harvested, RNA isolated and transcriptome analyzed using an oligonucleotide microarray platform. **B)** Edwards-Venn diagrams showing common or distinct regulated genes over the sampled time points. **C)** Clustering of differentially expressed genes. Clustering was performed using the Smooth correlation and average linkage clustering in GeneSpring GX 7.3.1 software (Agilent Technologies). Orange indicates up-regulated, green indicates down-regulated and white unchanged values, as shown on the color scale at the right side of the figure.

In order to demonstrate the efficiency of our system to elucidate early transcriptional responses, Arabidopsis seedlings were germinated and grown in the hydroponics system with 125 μM Pi during 12 days, and then immediately deprived of Pi. Samples were taken at three short-time points (10 min, 30 min, and 2 h) (Figure 8A). Roots were harvested and frozen immediately after each time point, total RNA extracted and their transcriptome analyzed by microarray expression profiling. For data analyses, differences in gene expression between Pi-depleted versus Pi-sufficient roots were identified (the overall P availability effect) and also the differences caused by the Pi availability by time interaction (time × Pi effect). According to the stringency levels used (FDR≤ 0.05 and fold ±2), a total of 181 genes showed differential expression in at least one of three sampled time points (see Additional file 3). A total of 92 genes were found to be up-regulated and 89 down-regulated by Pi-depletion (Figure 8B). Interestingly, only 3 genes out of the 92 induced and 1 down-regulated out of the 89 repressed were common to all three time points evaluated thus indicating specific transcriptional responses depending of the time point analyzed (Figure 8B). When clustered into functional classifications (Table 2 and Additional file 3), some resembled those previously reported [25-27,29], thus validating our system for high throughput transcriptional analyses. According to the expression profile, up-regulated genes were clustered in six different groups, whereas only three groups were identified for repressed genes (Additional file 3). Analysis of expression patterns by agglomerative hierarchical clustering showed a high number of up-regulated genes in the last time point evaluated (2 h) while an opposite tendency was observed for down-regulated genes which were more responsive in the first time point (10 min) (Figure 8C). Differentially expressed genes were classified into functional categories according to The Munich Information Center for Protein Sequences classification (MIPS) using the FunCat database [30]. Categories more represented in up-regulated genes were those related with Metabolism, Transcription, Protein metabolism, and Interaction with the environment (Table 2). Also, there was a similar number of induced and repressed genes in Pi, phospholipid, and phospholipid metabolism categories, with the exception of those related with glycolipid metabolism. Interestingly the Energy category (glycolysis, gluconeogenesis, pentose-phosphate pathway, respiration, energy conversion and regeneration, and light absorption) was only represented in induced genes (Table 2).

**Table 2:** Distribution of functional categories of differentially expressed genes responding to Pi-deprivation under short time points in Arabidopsis roots

Functional category*	Up-/Down-regulated genes** (%)		
	**Time point sampled**		
	**10 min**	**30 min**	**2 h**
Metabolism	9.67 /15.9	21.4/7.14	34.1/24.1
Energy	3.22/-	10.7/-	2.43/-
Cell cycle and DNA processing	3.22/2.27	3.57/7.14	4.87/6.89
Transcription	12.9/4.54	7.14/21.4	12.1/10.3
Protein fate	3.22/13.54	7.14/7.14	12.1/13.7
Protein with binding function or cofactor requirement	16.1/15.9	32.1/7.14	21.9/27.5
Regulation of metabolism and protein function	-	3.57/7.14	2.43/3.44
Cell transport, transport facilities, and transport routes	9.67/4.54	14.2/7.14	7.31/10.3
Cellular communication/signal transduction mechanism	-/2.27	-/14.2	-/3.44
Cell rescue, defense and virulence	3.22/2.27	10.7/14.2	9.75/10.3
Interaction with the environment	9.67/2.27	21.4/21.4	2.43/6.89
Systematic interaction with the environment	3.22/2.27	3.57/-	2.43/6.89
Cell fate	-/2.27	-	-
Development	6.45/2.27	-/14.2	-
Biogenesis of cellular components	-	3.57/7.14	2.43/6.89
Subcellular localization	29/34	35.7/21.4	26.8/34.4
Unclassified proteins	45.1/43.1	21.4/35.7	26.8/41.3

*Functional categories according to The Munich Information Center for Protein Sequences classification. **Differentially expressed genes with a fold change of at least ±2 at any time point and FDR ≤0.05.

#### *Early transcriptional responses to low Pi availability involves cell wall modifications, protein activity, oxidation-reduction processes, and hormones-mediated signaling that precede the reported Pi-signaling pathways*

According to the functional annotation of the Arabidopsis Information Resource database (TAIR, at http://www.arabidopsis.org), most genes, either induced or repressed during the first 30 min of Pi depletion, are related to cell wall composition, protein activity, oxidation-reduction, and hormones-mediated signaling. Previously known Pi-responsive genes such *MGDG SYNTHASE 3* (*MGD3*), *SQDG SYNTHASE 2* (*SQD2*), *PURPLE ACID PHOSPHATASE 22* (*PAP22*), and S*-ADENOSYLMETHIONINE SYNTHASE 1* (*SAM1*) presented significant changes in expression until the last time point evaluated (2 h). Interestingly, a few transcriptional controllers were expressed differentially throughout the entire experiment.

At 10 minutes, Arabidopsis roots responded to Pi-deprivation with the activation of 27 genes (18.5% of total) involved in polysaccharide degradation, callose deposition, pectin biosynthesis, cell expansion, and microtubule cytoskeleton organization (see group I, Additional file 3). Gene sets related with oxidation-reduction processes, protein activity modifications (ubiquitination, myristoylation, ATP or ion binding), and hormones-mediated signaling (abscisic acid, jasmonic acid) were also represented. Overrepresentation of groups according functional processes was not clear in down-regulated genes, excepting those related to modifications to protein fate (13.5% of total 44 genes).

As Pi depletion progressed (30 min), transcriptional changes related to cell wall decreased while responses to ion transport, signaling by hormones (auxins, abscisic acid, salicylic acid) or kinases were more represented in both induced and repressed genes (Additional file 3). In down-regulated genes, this trend was also found in the last time point (2 h). At 30 minutes, interestingly, genes involved with Pi-homeostasis, e.g. *SPX1* and *GLYCEROL-3-PHOSPHATE PERMEASE* 1 (*G3Pp1*), were already induced (see group IV and V, Additional file 3).

A higher number of up-regulated genes was found two hours after Pi-depletion. Most induced genes (9 out of 37 genes) were related to ion transport or homeostasis but also to carbohydrate metabolism, oxidation-reduction, signaling, protein activity and development. Importantly, other typical molecular markers for Pi starvation were also induced within 2 hours. Two phosphatidate phosphatases (PAPs) (At3g52820 and At5g44020) were induced gradually according Pi-starvation proceeded. *MGD3* and *SQD2*, both involved with Pi recycling, were also induced at 2 hours (see group VI, Additional file 3). Expression of these genes, together with *SPX1* and *G3Pp1*, indicate that the classical transduction pathways related with Pi-starvation can be triggered as early as two hours after seedlings are exposed to media lacking Pi. *SPX1* is strongly induced by Pi starvation and usually classified as member of a system signaling pathway depending of SIZ1/PHR1 reviewed in [31]. Its early induction (3–12 h) has been previously reported [25] however an “immediate-early response” within few minutes after Pi depletion has been not reported so far. Likewise, a role for an enhanced expression of *G3Pp1* inside transduction pathways or metabolic rearrangements triggered by Pi stress is still poorly understood [25,26]. A recent functional characterization of Arabidopsis glycerophosphodiester phosphodiesterase (GDPD) family suggests glycerol-3-phosphate (G3P) as source of Pi or phosphatidic acid (PA), which could be used by glycerol-3 phosphatase (GPP) or DGDG/SQDG pathways [32]. Early induced expressions of *G3Pp1*, *PAP22*, and *MGD3* is in agreement with the hypothesis that under Pi deficiency G3P could be first converted into PA by two acyltransferase reactions and Pi would be then released during the subsequent conversion of PA into diacylglycerol (DAG) by PAPs [32]. DAG produced could be incorporated into DGDG or SQDG by MGD2/3 and DGD1/2 and SQD1/2, respectively [32]. *MGD2* and *MGD3* have been found induced in Arabidopsis seedlings depleted of Pi for 3–12 h [25]. This early transcriptional activity for *MGD* genes during Pi starvation is also reflected in enhanced enzymatic activities as revealed in Pi-starved bean roots [33]. Increased PA levels and MGDG and DGDG activities have been reported in bean roots starved of Pi for less than 4 h [31]. Early gene expression activation of genes encoding MGDG and DGDG but not PLD/C enzymes suggests G3P and not PC as source for PA and DAG biosynthesis for early Pi signaling and recycling pathways.

According with our data, a specific transduction pathway to Pi deficiency could be preceded by general responses related to stress, which could modify metabolism before triggering specific expression of transcriptional factors. This idea is consistent with previous reports assaying Pi-depletion in Arabidopsis by short and medium-long times (3–48 h), which also reported differentially expressed genes related with pathogenesis, hormone-mediated signaling, protein activity, redox processes, ion transport, and cell wall modifications [25,28,34]. Similar results have been recently reported in rice seedlings under Pi starvation for 1 h [35].

### Applications of our hydroponic system: 2) Pathological assays to evaluate systemic defense responses

**Figure 9:**
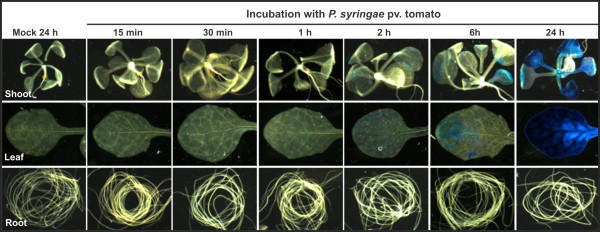
**PR1::GUS expression pattern under *P. syringae *pv. tomato incubation.** Arabidopsis PR1::GUS seedlings were grown hydroponically and then transferred to liquid media containing *P. syringae* bacteria (final 0.002 OD_600)_ or control media (mock). GUS activity in shoot and root was monitored by histochemical analyses at different time points. GUS expression in mock condition is shown for the last time point sampled (24 h).

In order to determine the suitability of our hydroponic system to perform Arabidopsis-pathogen interactions, we evaluated the systemic effect of root inoculation with *Pseudomonas syringae* pv *tomato* strain DC3000 (*Pst* DC3000) on transcriptional activation of the pathogenesis-related gene *PR1*. Although *P. syringae* is generally known as a leaf pathogen, it has been proven to be an excellent root colonizer in Arabidopsis [36,37]. Transgenic Arabidopsis seedlings carrying the *PR1::GUS* construct were grown for 12 days and then inoculated with 0.002 OD_600_ of fresh bacterial inoculum. β-glucoronidase (GUS) activity was analyzed by histochemical staining at different time intervals after inoculation. Systemic response to *Pst* root colonization was evident between 2 and 6 hours after inoculation (hai), as revealed by strong expression of the marker gene in leaves. After 24 hai, GUS activity spread throughout the whole shoot system, but not in roots (Figure 9). These results demonstrate a good performance for studying plant responses to pathogens.

## Conclusions

Here, we describe a practical and inexpensive hydroponic system for growing Arabidopsis and other plants under sterile conditions with an in vitro growth window that goes from seedlings to adult plants. Our system uses recyclable and plastic materials sterilizable by conventional autoclaving that are easy to get at local markets. In contrast to other hydroponic systems previously reported, the components of the system (container size, mesh density, lid) described here can be easily adapted to different experimental designs or plant species. The seed-holder avoids the use of an agarose plug or any other accessory reducing time for setting up experiments and decreasing risks of contamination.

Applications and advantages of our hydroponic system are exemplified in this report. First, rapid transcriptome changes of Arabidopsis roots induced by Pi depletion were detected by a rapid harvest from growth media using our new seed-holder designed. Our analyses confirm that Arabidopsis roots early responses to Pi depletion includes the activation of signaling pathways related to general stress before to trigger those specific to Pi stress, and support the idea that G3P could be a source of Pi and other molecules as PA during early signaling events induced by Pi starvation. Second, our hydroponic system showed a high performance to set up pathogenesis assays.

## Methods

### Growth media

For solid or liquid media, a 0.1 X Murashige and Skoog (MS) medium, pH 5.7, supplemented with 0.5% sucrose (Sigma-Aldrich), and 3.5 mM MES (Sigma-Aldrich) was used. For solid growth media, agar plant TC micropropagation grade (composition/purity not provided) (A296, Phytotechnology Laboratories, US) from *Gelidium* species was used at 1% (W/V).

### Plant material and growth conditions

*Arabidopsis thaliana* ecotype Columbia (Col-0, ABRC stock No. 6000 from SALK Institute), marker lines PR1::GUS (At2g14610) (kindly provided by F.M. Ausubel) and AtPT2::GUS (At2g38940) [38], tobacco cv Xhanti, tomato cv Micro-Tom, and *Setaria viridis* seeds were used in this study. In all cases, seeds were surface sterilized by sequential treatments with absolute ethanol for 7 min, 20% (v/v) commercial bleach for 7 min and rinsed three times with sterile distilled water. Previous sterilization, *Setaria* seeds were placed at -80°C overnight as recommended [39]. Arabidopsis sterilized seeds were vernalized at 4°C for 2 days (solid media) or 4 days (hydroponics). For Arabidopsis, 50–65 seeds were directly sowed on the mesh of the seed-holder, and 30–35 seeds in Petri plates (50 ml volume, 15 mm × 150 mm, Phoenix Biomedical). Seedlings of all species evaluated were grown at 22°C, except tobacco which was grown at 28°C, using growth chambers (Percival, Perry, IA, USA) with fluorescent light (100 μmol m^-2^ s^-1^) and a photoperiod of 16 h light/8 h dark.

### Analysis of root architecture traits

To determine root architecture traits, seedlings were grown on agar plates at an angle of 65° or under hydroponic conditions. Root length was measured from root tip to hypocotyls base. For lateral root (LR) quantification, all clearly visible emerged secondary roots were taken into account when the number of LRs was determined. Root hair density was calculated from root images taken with a digital camera connected to an AFX-II-A stereomicroscope (Nixon, Tokyo). Statistical analysis of quantitative data was performed using the statistical tools (Student’s t test) of Microsoft Excel software.

### GUS analyses

For histochemical analysis of GUS activity, *Arabidopsis* seedlings were incubated for 4 h at 37°C in a GUS reaction buffer (0.5 mg ml^-1^ of 5-bromo-4-chloro-3-indolyl-b-D-glucuronide in 100 mM sodium phosphate buffer, pH 7). Seedlings were cleared using the method previously described [40]. At least 15 transgenic plants were analyzed and imaged using Normarski optics on a Leica DMR microscope.

### Microarray analysis

Roots were collected and immediately frozen and RNA isolated using the Trizol reagent (Invitrogen) and purified with the RNeasy kit (Qiagen) following the manufacturer’s instructions. Spotten glass microarray slides (Arabidopsis Oligonucleotide Array version 3.0) were obtained from University of Arizona (http://.ag.arizona.edu/microarray/). Three biological replicates (150 seedlings per replicate) were used for RNA isolation and two technical replicates (in swap) to the two channel microarrays. Fluorescent labeling of probes, slide hybridization, washing, and image processing was performed as described [27]. A loop design was used in order to contrast the gene expression differences between treatments and time points. Microarray normalization and data analysis to identify differentially expressed genes with at least two-fold change in expression were carried as previously reported [27].

The microarray data have been deposited in Gene Expression Omnibus (GEO) and are accessible through GEO Series, accession number: GSE53114 (http://www.ncbi.nlm.nih.gov/geo/query/acc.cgi?acc=GSE53114).

### Transcript analysis

Total RNA was extracted with Trizol (Invitrogen) and purified using Qiagen RNeasy columns according to the manufacturer’s instructions. cDNA was synthesized using 30 μg of total RNA with SuperScript III Reverse Transcriptase (Invitrogen) and used for qRT-PCR (7500 Real Time PCR System, Applied Biosystems). Expression of marker genes for Pi starvation was analyzed using the oligonucleotides listed in the Additional file 4. Gene expression analyses were performed as previously reported [27]. Briefly, Relative quantification number (RQ) was obtained from the equation (1 + E)2^∆∆C^_T_ where ∆∆CT represents ∆CT (Treatment)-∆CT (Control) and E is the PCR efficiency. Each CT was previously normalized using the expression levels of ACT2 (At3g18780), PPR (At5g55840), and UPL7 (At3g53090) as internal references.

### *Pseudomonas syringae* bioassays

Bacterial strain *P. syringae* pv *tomato* (*Pst*) DC3000 was cultured on King*’*s B medium (KB) supplied with 50 μg ml^-1^ rifampicin. *PR1::GUS* seedlings were grown hydroponically as described above. For infection of 12 day-old seedlings, a bacterial culture was grown overnight in KB at 28°C. Bacteria were centrifuged, washed three times with sterilized water and resuspended in water to a final OD_600_ of 0.04. The appropriate volume was added to seedling medium to a final OD_600_ of 0.002. After infection, plant material was harvested at several time points for GUS histochemical assays and analyzed under a Leica DMR microscope.

## Abbreviations

MGDG: Monogalactosyldiacylglycerol; DGDG: Digalactosyldiacylglycerol; SQDG: Sulfoquinovosyldiacylglycerol.

## Competing interests

The authors declare that they have no competing interests.

## Authors’ contributions

FAC, CCV and LHE conceived the new hydroponic system and designed the experiments. FAC, CCV, EIL, LYV, C-APT, AOA, AMB, S-IGM, DAG, and B-APO performed the experiments. FAC, CCV, EIL, and LHE analyzed the data. FAC, CCV, and LHE drafted the manuscript. All authors read and approved the final manuscript.

## Supplementary Material

Additional file 1 **35-40-day-old Arabidopsis plants growing under our hydroponic system proposed.** The Arabidopsis seeds were directly sowed on the seed-holder and three adult plants per vessel were grown until the flowering began. Click here for file

Additional file 2 Video showing the assembly process for our hydroponic system. Click here for file

Additional file 3 Differentially expressed genes by Pi depletion during short time points in Arabidopsis roots. Click here for file

Additional file 4 qRT-PCR primers used in this study. Click here for file
