# Supplementary material for: An improved, low-cost, hydroponic system for growing Arabidopsis and other plant species under aseptic conditions
Source: BMC Plant Biol. 2014 Mar 21;14:69. doi: 10.1186/1471-2229-14-69 (PMC3999955; doi:10.1186/1471-2229-14-69)

Additional file 1. 35-40-day-old Arabidopsis plants growing under our hydroponic system proposed. The Arabidopsis seeds were directly sowed on the seed-holder and three adult plants per vessel were grown until the flowering began.

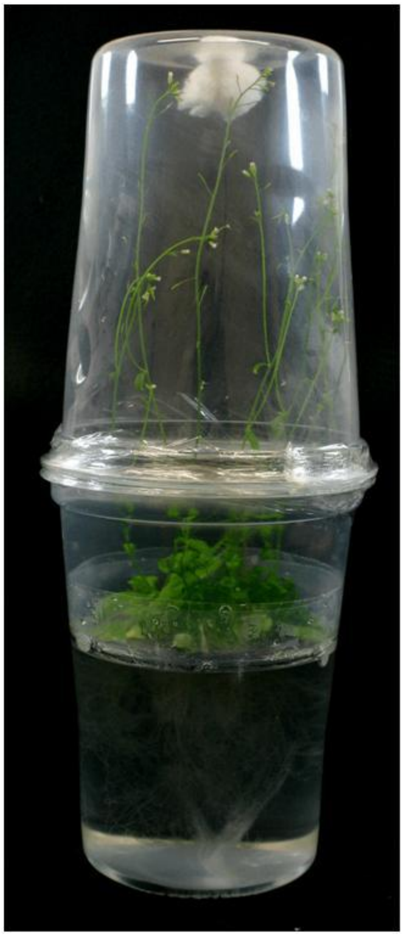

Supplement: Additional file 1 — 35-40-day-old Arabidopsis plants growing under our hydroponic system proposed. The Arabidopsis seeds were directly sowed on the seed-holder and three adult plants per vessel were grown until the flowering began. [file 1471-2229-14-69-S1.pdf]
