# Supplementary material for: An improved, low-cost, hydroponic system for growing Arabidopsis and other plant species under aseptic conditions
Source: BMC Plant Biol. 2014 Mar 21;14:69. doi: 10.1186/1471-2229-14-69 (PMC3999955; doi:10.1186/1471-2229-14-69)
Supplement: Additional file 3 — Differentially expressed genes by Pi depletion during short time points in Arabidopsis roots. [file 1471-2229-14-69-S3.pdf]

Additional file 3. Differentially expressed genes by Pi depletion during short time points in Arabidopsis roots.

UP-REGULATED GENES					
**ID (array)**	**Description**	**Expression ratio** 10 min	30 min	2 h	**Biological process involved**
**Group I**					
At1g01980	FAD-binding domain-containing protein similar to SP|P30986 reticuline oxidase precursor (Berberine-bridge-forming enzyme) (BBE) (Tetrahydroprotoberberine synthase) [Eschscholzia californica]; contains PF01565 FAD binding domain	2.0571	1.0405	1.2171	Oxidation-reduction process
At1g20150	Subtilase family protein similar to subtilisin-type protease precursor GI:14150446 from [Glycine max]	2.2412	1.1103	0.9798	Proteolysis, Defense-related gene (rice)
At1g21970	CCAAT-box binding transcription factor (LEC1) similar to CAAT-box DNA binding protein subunit B (NF-YB) (SP:P25209) (GI:22380) [Zea mays]; identical to GB:AAC39488 GI:3282674 from [Arabidopsis thaliana] (Cell 93 (7), 1195-1205 (1998)); identified in Plant	2.0464	1.0286	1.0259	ABA-mediated signaling pathway
At1g56660	Expressed protein	2.0959	0.9956	1.0881	---
At1g62000	Expressed protein	2.1127	1.6064	0.9992	---
At1g64100	Pentatricopeptide (PPR) repeat-containing protein contains Pfam profile PF01535: PPR repeat	4.2749	1.1753	1.2082	Transcription
At1g66250	Glycosyl hydrolase family 17 protein similar to beta-1,3-glucanase GI:15150341 from [Camellia sinensis]	2.122	1.0356	1.3966	Cell wall metabolism
At1g66450	DC1 domain-containing protein contains Pfam profile PF03107: DC1 domain	2.0199	1.2472	0.9931	Oxidation-reduction process, Cytokinin biosynthetic pathway
At1g68790	Expressed protein	2.045	1.3335	1.0114	Microtubule cytoskeleton organization
At2g22610	Kinesin motor protein-related	2.6697	1.3129	1.3415	Microtubule cytoskeleton organization
At2g22795	Expressed protein	2.3468	1.0586	0.9482	---
At2g29890	Villin 1 (VLN1) nearly identical to villin 1 (VLN1) [Arabidopsis thaliana] GI:3415113	2.9965	1.4006	0.9295	Cell expansion
At3g05440	C2 domain-containing protein similar to cold-regulated gene SRC2 [Glycine max] GI:2055230; contains Pfam profile PF00168: C2 domain	2.1089	1.286	0.9741	Response to cold (soybean)
At3g05900	Neurofilament protein-related similar to NF-180 (GI:632549) [Petromyzon marinus] similar to Neurofilament triplet H protein (200 kda neurofilament protein) (Neurofilament heavy polypeptide) (NF-H) (Swiss-Prot:P12036) [Homo sapiens]	2.1615	1.094	1.2476	Cell wall metabolism
At3g18120	F-box family protein-related contains TIGRFAM TIGR01640 : F-box protein interaction domain; similar to F-box protein family, atfbx9 (GI:20197985) [Arabidopsis thaliana]	3.1488	1.0907	1.0364	Ubiquitination
At3g28580	AAA-type ATPase family protein contains Pfam profile: ATPase family PF00004	2.327	1.0655	1.0595	Response to ABA and ethylene
At4g25210	Expressed protein weak similarity to storekeeper protein [Solanum tuberosum] GI:14268476; contains Pfam profile PF04504: Protein of unknown function, DUF573	2.5187	0.9301	0.9193	Transcription
At4g31660	Transcriptional factor B3 family protein low similarity to reproductive meristem gene 1 from [Brassica oleracea var. Botrytis] GI:3170424; contains Pfam profile PF02362: B3 DNA binding domain	2.0743	0.9575	1.0684	---

At5g15620	F-box family protein contains F-box domain Pfam:PF00646	3.5662	1.2191	1.0718	Response to water stress
At5g17260	No apical meristem (NAM) family protein contains Pfam PF02365: No apical meristem (NAM) domain;	2.1029	0.7698	0.8915	Transcription
At5g21970	Expressed protein supported by full-length cdna gi:22531216 from [Arabidopsis thaliana]	2.1253	1.0059	0.7792	Ubiquitination
At5g23960	Terpene synthase/cyclase family protein non-consensus TA donor splice site at exon 4	2.2779	1.3361	1.1005	Response to herbivore and bacteria, sesquiterpene biosynthesis
At5g38720	Expressed protein predicted protein, Drosophila melanogaster	2.2148	1.1651	0.8386	---
At5g40450	Expressed protein	3.2814	1.4503	1.26	Flavonoid biosynthesis
At5g55660	Expressed protein similar to unknown protein (pir||T08929)	2.2227	0.9991	1.0339	---
At5g60630	Expressed protein predicted protein, Arabidopsis thaliana	2.2148	1.1565	0.9995	---
At5g63550	Expressed protein	2.0248	0.9768	1.1095	---
**Group II**					
At3g22120	Protease inhibitor/seed storage/lipid transfer protein (LTP) family protein similar to SP|Q00451|PRF1_LYCES 36.4 kda proline-rich protein Lycopersicon esculentum, proline-rich cell wall protein [Medicago sativa] GI:3818416; contains Pfam profile PF00234 P	2.145	2.2295	0.7955	Cell wall metabolism
At1g77120	Alcohol dehydrogenase (ADH) identical to alcohol dehydrogenase GI:469467 from (Arabidopsis thaliana)	2.1016	2.2782	0.9848	Cell respiration, oxidation-reduction process
**Group III**					
At5g10040	Expressed protein	2.4727	2.9128	2.9882	Cell respiration, SA-mediated signaling pathway
At2g14247	Expressed protein	2.2689	1.6019	2.6504	---
**Group IV**					
At1g04540	C2 domain-containing protein low similarity to cold-regulated gene SRC2 [Glycine max] GI:2055230; contains Pfam profile PF00168: C2 domain	1.2372	2.1895	1.3655	---
At1g07610	Metallothionein-like protein 1C (MT-1C) identical to Metallothionein-like protein 1C (MT-1C). (SP:Q38804) (Arabidopsis thaliana)	0.9576	2.205	0.7919	Ion transport
At1g43260	Hypothetical protein contains Pfam domain, PF04937: Protein of unknown function (DUF 659)	0.9113	2.1256	1.3626	Transposon
At1g61520	Chlorophyll A-B binding protein / LHCI type III (LHCA3.1) nearly identical to PSI type III chlorophyll a/b-binding protein GI:430947; contains Pfam profile: PF00504 chlorophyll A-B binding protein; similar to PSI type III chlorophyll a/b-binding protein G	1.4043	2.3291	0.8539	Photosynthesis
At2g04135	Hypothetical protein	1.0158	2.8943	0.8432	---
At2g12280	Ligase, putative identical to residues 550 - 623 of 10-formyltetrahydrofolate synthetase (Formate--tetrahydrofolate ligase (EC 6.3.4.3)) from Arabidopsis thaliana GI:5921663	1.1024	2.0946	0.5998	Folic acid metabolism
At2g25200	Expressed protein	0.822	2.0942	1.116	---
At2g28860	Cytochrome P450 family protein similar to Cytochrome P450 61 (C-22 sterol desaturase) (SP:P54781) {Saccharomyces cerevisiae}	1.0938	2.4151	0.5886	Oxidation-reduction process
At2g39730	Ribulose bisphosphate carboxylase/oxygenase activase / rubisco activase identical	1.4878	2.5154	1.2095	Photosynthesis

	to SWISS-PROT:P10896 ribulose bisphosphate carboxylase/oxygenase activase, chloroplast precursor (rubisco activase, RA)[Arabidopsis thaliana]				
At3g03847	Auxin-responsive family protein similar to auxin-inducible SAUR (Small Auxin Up rnas) (GI:3043536) [Raphanus sativus]	0.9949	2.4934	0.9264	Response to auxin stimulus
At3g12580	Heat shock protein 70, putative / HSP70, putative strong similarity to heat shock protein GI:425194 [Spinacia oleracea]	0.9796	3.261	0.5339	Proteim metabolism, ion transport, response to bacteria and heat
At3g21890	Zinc finger (B-box type) family protein contains Pfam profile: PF01760 CONSTANS family zinc finger	0.7663	2.2384	0.7268	Transcription, response to UV-B
At3g55800	Sedoheptulose-1,7-bisphosphatase, chloroplast / sedoheptulose-bisphosphatase identical to SP|P46283 Sedoheptulose-1,7-bisphosphatase, chloroplast precursor (EC 3.1.3.37) (Sedoheptulose-bisphosphatase) (SBPASE) (SED(1,7)P2ASE) {Arabidopsis thaliana}	1.4279	2.4922	1.0561	MAPK cascade, carbohydrate metabolism
At3g56290	Expressed protein	1.0792	2.0388	1.171	Transcription
At4g25440	WD-40 repeat family protein / zfwd1 protein (ZFWD1) identical to zfwd1 protein (GI:12057164) [Arabidopsis thaliana]	1.0633	2.0549	1.2079	Ion transport
At4g28750	Photosystem I reaction center subunit IV, chloroplast, putative / PSI-E, putative (PSAE1) identical to SP|Q9S831; similar to SP|P12354 Photosystem I reaction center subunit IV, chloroplast precursor (PSI-E) {Spinacia oleracea}; contains Pfam profile PF024	1.0074	2.3766	0.6277	Photosynthesis
At4g37550	Formamidase, putative / formamide amidohydrolase, putative similar to SP|Q50228 Formamidase (EC 3.5.1.49) (Formamide amidohydrolase) {Methylophilus methylotrophus}; contains Pfam profile PF03069: Acetamidase/Formamidase family	1.2817	2.025	0.8268	---
At5g20150	SPX (SYG1/Pho81/XPR1) domain-containing protein similar to PHO1 protein [Arabidopsis thaliana] GI:20069032; contains Pfam profile PF03105: SPX domain	1.0305	3.79	1.2851	Pi transport and signaling pathway
At5g38420	Ribulose bisphosphate carboxylase small chain 2B / rubisco small subunit 2B (RBCS-2B) (ATS2B) identical to SP|P10797 Ribulose bisphosphate carboxylase small chain 2B, chloroplast precursor (EC 4.1.1.39) (rubisco small subunit 2B) {Arabidopsis thaliana}	1.0316	2.2434	1.0168	Photosynthesis
At5g42720	Glycosyl hydrolase family 17 protein similar to glucan endo-1,3-beta-glucosidase precursor SP:P52409 from [Triticum aestivum]	1.0865	2.0064	0.9299	Carbohydrate metabolism
At5g51190	AP2 domain-containing transcription factor, putative contains similarity to ethylene responsive element binding factor	1.2517	2.048	0.9006	Transcription, response to chitin, mechanical stimulus and wounding
At5g52640	Heat shock protein 81-1 (HSP81-1) / heat shock protein 83 (HSP83) nearly identical to SP|P27323 Heat shock protein 81-1 (HSP81-1) (Heat shock protein 83) {Arabidopsis thaliana}; contains Pfam profiles PF02518: ATPase, histidine kinase-, DNA gyrase B-, and	1.0446	2.7368	0.7718	Proteim metabolism, ion transport response to bacteria and heat
**Group V**					
At3g47420	Glycerol-3-phosphate transporter, putative / glycerol 3-phosphate permease, putative similar to camp inducible 2 protein [Mus musculus] GI:4580997, glycerol-3-phosphate transporter (glycerol 3-phosphate permease) [Homo sapiens] GI:7543982; contains Pfam p	1.078	3.3258	2.8003	Pi homeostasis
At5g42530	Expressed protein	1.5563	3.9044	2.0844	---
**Group VI**					
At1g02500	S-adenosylmethionine synthetase 1 (SAM1) identical to S-adenosylmethionine	1.1695	1.4747	4.0156	Ion transport, response to salt stress,

	synthetase 1 (Methionine adenosyltransferase 1, adomet synthetase 1) [Arabidopsis thaliana] SWISS-PROT:P23686				temperatura and wounding
At1g14550	Anionic peroxidase, putative similar to anionic peroxidase GI:170202 from (Nicotiana sylvestris)	0.9398	0.8465	2.2612	Oxidative stress
At1g21110	O-methyltransferase, putative similar to GI:2781394	1.0285	0.618	2.0757	---
At1g22830	Pentatricopeptide (PPR) repeat-containing protein contains Pfam profile PF01535: PPR repeat. Gene continues on the 3' end of BAC F19G10 gb|AF000657 gene F19G10.21	1.2866	1.1052	2.4555	---
At1g24510	T-complex protein 1 epsilon subunit, putative / TCP-1-epsilon, putative / chaperonin, putative identical to SWISS-PROT:O04450- T-complex protein 1, epsilon subunit (TCP-1-epsilon) [Arabidopsis thaliana]; strong similarity to SP|P54411 T-complex protein 1,	1.3295	0.9452	2.071	Protein metabolism, RNA processing
At1g49930	Hypothetical protein	0.9694	1.4515	2.1928	---
At1g50640	Ethylene-responsive element-binding factor 3 (ERF3) identical to SP|O80339 Ethylene responsive element binding factor 3 (aterf3) [Arabidopsis thaliana]	1.0249	1.0753	2.1262	Response to salt stress, ethylene
At1g61890	MATE efflux family protein similar to ripening regulated protein DDTFR18 [Lycopersicon esculentum] GI:12231296; contains Pfam profile PF01554: Uncharacterized membrane protein family	1.3738	0.8671	2.1002	Drug transmembrane transport, response to ABA and JA
At1g65310	Xyloglucan:xyloglucosyl transferase, putative / xyloglucan endotransglycosylase, putative / endo-xyloglucan transferase, putative similar to xyloglucan endotransglycosylase TCH4 GI:886116 from [Arabidopsis thaliana]	0.8885	1.1198	2.1177	Carbohydrate metabolism
At1g72200	Zinc finger (C3HC4-type RING finger) family protein contains Pfam profile: PF00097: Zinc finger, C3HC4 type (RING finger)	0.8005	1.042	2.0836	Iron starvation, nitrate transport
At1g78830	Curculin-like (mannose-binding) lectin family protein similar to S glycoprotein [Brassica rapa] GI:2351186; contains Pfam profile PF01453: Lectin (probable mannose binding)	0.845	1.1101	2.0994	Sugar binding
At1g80380	Phosphoribulokinase/uridine kinase-related	0.9276	0.5884	2.1529	Photorespiration
At2g11810	1,2-diacylglycerol 3-beta-galactosyltransferase, putative / monogalactosyldiacylglycerol synthase, putative / MGDG synthase, putative identical to monogalactosyldiacylglycerol synthase type C [gi:9927295] from Arabidopsis thaliana, similar to MGDG synthas	0.7615	0.9166	2.0534	Cellular response to Pi starvation
At2g27600	AAA-type ATPase family protein / vacuolar sorting protein-related similar to SP|P46467 SKD1 protein (Vacuolar sorting protein 4b) {Mus musculus}; contains Pfam profiles PF00004: ATPase AAA family, PF04212: MIT domain	1.029	0.9004	2.2814	Endosomal transport, lipid transport, fatty acid beta-oxidation
At2g29750	UDP-glucoronosyl/UDP-glucosyl transferase family protein contains Pfam profile: PF00201 UDP-glucoronosyl and UDP-glucosyl transferase	0.9777	1.1907	2.7215	Iron starvation, nitrate transport, cell root differentiation
At2g39510	Nodulin mtn21 family protein similar to mtn21 GI:2598575 (root nodule development) from [Medicago truncatula]	1.5245	1.2307	2.1967	--
At2g42250	Cytochrome P450 family protein similar to cytochrome P450 93A1 (SP:Q42798) [Glycine max]	0.9391	1.1	2.1655	Oxidation-reduction process
At3g20050	T-complex protein 1 alpha subunit / TCP-1-alpha / chaperonin (CCT1) identical to SWISS-PROT:P28769- T-complex protein 1, alpha subunit (TCP-1-alpha) [Arabidopsis thaliana]	1.4807	0.657	2.2397	Cytoskeleton organization, RNA methylation, protein metabolism
At3g28150	Expressed protein	1.2303	1.1593	2.0143	Carbohydrate metabolism
At3g28350	Hypothetical protein	1.1961	1.0934	2.0949	---
At3g46600	Scarecrow transcription factor family protein scarecrow-like 11 - Arabidopsis thaliana, EMBL:AF036307	1.0472	0.8991	2.3635	Oxidation-reduction process
At3g47050	Glycosyl hydrolase family 3 protein beta-D-glucan exohydrolase, Nicotiana tabacum,	0.8134	1.4756	2.0458	Carbohydrate metabolism

	TREMBL:AB017502_1				
At3g52820	Purple acid phosphatase (PAP22) identical to purple acid phosphatase (PAP22)GI:20257494 from [Arabidopsis thaliana]	0.8651	1.3859	2.5724	Cellular response to Pi starvation
At3g57520	Alkaline alpha galactosidase, putative similar to alkaline alpha galactosidase II [Cucumis melo] GI:29838631; contains Pfam profile PF05691: Raffinose synthase or seed imbibition protein Sip1	1.415	0.8368	2.1679	Carbohydrate metabolism, oxidative stress
At3g60550	Cyclin family protein similar to cyclin 2 [Trypanosoma brucei] GI:7339572, cyclin 6 [Trypanosoma cruzi] GI:12005317; contains Pfam profile PF00134: Cyclin, N-terminal domain	1.0758	1.2071	2.6179	Cell cycle
At4g15630	Integral membrane family protein contains TIGRFAM TIGR01569 : plant integral membrane protein TIGR01569; contains Pfam PF04535 : Domain of unknown function (DUF588)	0.7925	1.154	2.4257	---
At4g38220	Aminoacylase, putative / N-acyl-L-amino-acid amidohydrolase, putative similar to aminoacylase-1 (N-acyl-L-amino-acid amidohydrolase, ACY-1)[Homo sapiens] SWISS-PROT:Q03154	1.4328	1.3394	2.3245	Protein metabolism, response to zinc ion
At5g01220	UDP-sulfoquinovose:DAG sulfoquinovosyltransferase / sulfolipid synthase (SQD2) identical to GI:20302857	1.1487	0.8449	2.7589	Cellular response to Pi starvation
At5g09860	Nuclear matrix protein-related low similarity to nuclear matrix protein p84 [Homo sapiens] GI:550058	1.1899	1.123	2.2289	RNA metabolism
At5g13680	IKI3 family protein weak similarity to SP|O95163 ikappab kinase complex-associated protein (IKK complex-associated protein) (p150) {Homo sapiens}; contains Pfam profile PF04762: IKI3 family	1.4893	1.0278	2.3403	Cell proliferation, response to ABA and oxidative stress
At5g15180	Peroxidase, putative similar to peroxidase ATP12a [Arabidopsis thaliana] gi|1429217|emb|CAA67311	0.8084	0.7798	2.2551	Oxidation-reduction process
At5g20790	Expressed protein predicted protein, Arabidopsis thaliana	0.8237	1.1518	2.2024	---
At5g44020	Acid phosphatase class B family protein similar to SP|P15490 STEM 28 kda glycoprotein precursor (Vegetative storage protein A) {Glycine max}, acid phosphatase [Glycine max] GI:3341443; contains Pfam profile PF03767: HAD superfamily (subfamily IIIB) phosph	0.8098	0.9019	2.1261	Celular cation homeostasis
At5g54680	Basic helix-loop-helix (bhlh) family protein similar to unknown protein (pir |B71406)	0.9241	1.0171	2.7488	Transcription
At5g60850	Dof-type zinc finger domain-containing protein similar to zinc finger protein OBP4 gi:5059396 from [Arabidopsis thaliana]; EMBL:AF155817	1.0971	1.1676	2.2409	Transcription
At5g63570	Glutamate-1-semialdehyde 2,1-aminomutase 1 (GSA 1) / glutamate-1-semialdehyde aminotransferase 1 (GSA-AT 1) identical to GSA 1 [SP|P42799]	1.2606	1.1754	2.8875	Aromatic amino acid biosynthesis, cell diferentiation
At5g65207	Expressed protein	0.751	0.871	2.8645	---

DOW-REGULATED GENES

ID (array)	Description	Expression ratio			Biological process involved
		10 min	30 min	2 h	
**Group I**					
At1g06750	Hypothetical protein	0.4815	0.6013	0.738	Cell wall modification
At1g12540	Basic helix-loop-helix (bhlh) family protein contains Pfam profile: PF00010 helix-loop-	0.4825	1.0873	0.8914	Transcription

	helix DNA-binding domain				
At1g29460	Auxin-responsive protein, putative similar to auxin-induced protein 6B (SP:P33083) [Glycine max]	0.346	1.0293	0.8982	Response to auxin stimulus
At1g52790	Oxidoreductase, 2OG-Fe(II) oxygenase family protein similar to GS-AOP loci [GI:16118889, GI:16118887, GI:16118891, GI:16118893]; contains PF03171 2OG-Fe(II) oxygenase superfamily domain	0.3966	1.0589	0.7218	Oxidation-reduction process
At1g56270	Hypothetical protein	0.4308	1.0735	1.0735	---
At1g63105	Hypothetical protein	0.4668	0.9165	0.9107	---
At1g66640	Expressed protein	0.4089	1.3264	0.9445	---
At1g69610	Expressed protein	0.4783	1.0476	0.8177	Protein metabolism
At1g70260	Nodulin mtn21 family protein contains similarity to mtn21 [Medicago truncatula] GI:2598575; contains Pfam profile PF00892: Integral membrane protein	0.4231	0.862	0.7688	---
At1g70960	F-box family protein contains Pfam PF00646: F-box domain; contains TIGRFAM TIGR01640: F-box protein interaction domain	0.4342	0.8453	0.5789	---
At1g76620	Expressed protein contains Pfam profile PF04784: Protein of unknown function, DUF547	0.488	1.0653	0.9654	---
At1g77655	Expressed protein	0.4055	1.2536	0.8027	---
At1g78915	Expressed protein	0.362	0.829	0.939	---
At2g03770	Sulfotransferase family protein similar to steroid sulfotransferase 3 [Brassica napus] GI:3420008; contains Pfam profile PF00685: Sulfotransferase domain	0.4796	0.9117	0.855	Flavonoid metabolism
At2g04800	Expressed protein	0.4227	1.112	0.7233	Protein metabolism, response to iron starvation and transport, response to nitrate
At2g06630	Hypothetical protein similar to At2g12120, At1g45090, At2g05470, At5g28482	0.498	0.8734	0.7775	Transposon, cell death
At2g17430	Seven transmembrane MLO family protein / MLO-like protein 7 (MLO7) identical to membrane protein Mlo7 [Arabidopsis thaliana] gi|14091584|gb|AAK53800; similar to MLO protein SWISS-PROT:P93766, NCBI_gi:1877221 [Hordeum vulgare][Barley]	0.3871	0.6384	0.8699	Cell death, pollen tube reception
At2g25470	Leucine-rich repeat family protein contains leucine rich-repeat (LRR) domains Pfam:PF00560, INTERPRO:IPR001611; contains similarity to disease resistance protein [Lycopersicon esculentum] gi|3894383|gb|AAC78591	0.4918	1.0168	0.8146	Signal transduction
At2g27320	Hypothetical protein contains Pfam profile PF03080: Arabidopsis proteins of unknown function	0.468	1.1177	0.8169	---
At2g28960	Leucine-rich repeat protein kinase, putative similar to light repressible receptor protein kinase [Arabidopsis thaliana] gi|1321686|emb|CAA66376; contains leucine rich repeat (LRR) domains, Pfam:PF00560; contains protein kinase domain, Pfam:PF00069	0.4938	0.8409	0.9565	ATP binding, response to iron starvation, kinase activity
At2g42630	Protein kinase family protein contains protein kinase domain, Pfam:PF00069	0.4948	1.0771	0.9031	Kinase activity
At3g23420	F-box family protein-related contains TIGRFAM TIGR01640: F-box protein interaction domain	0.4126	1.03	0.9925	---
At3g27410	Expressed protein	0.3549	0.818	0.8513	---
At3g50760	Glycosyl transferase family 8 protein contains Pfam profile: PF01501 Glycosyl transferase family 8	0.4966	0.9449	0.9442	Carbohydrate metabolism, JA, biosynthesis, response to fungus and wounding
At4g05630	Expressed protein	0.3084	0.8873	0.8243	---
At4g08130	Hypothetical protein	0.4449	0.8513	0.5458	---
At4g14250	UBX domain-containing protein low similarity to 60S ribosomal protein L2 [Nicotiana tabacum] GI:9230281; contains Pfam profile PF00789: UBX domain	0.4197	0.8489	0.8785	Ubiquitination
At4g14290	Expressed protein contains Interpro entry IPR000379	0.3009	0.739	0.6914	Response to abiotic stress

At4g15100	Serine carboxypeptidase S10 family protein similar to Serine carboxypeptidase II chains A and B (SP:P08819) (EC 3.4.16.6) [Triticum aestivum (Wheat)]	0.4959	1.1485	0.7751	Proteolysis, response to fungus
At4g25400	Basix helix-loop-helix (bhlh) family protein contains Pfam profile: PF00010 helix-loop-helix DNA-binding domain	0.4833	0.7922	0.9993	Transcription
At4g32915	Expressed protein	0.4814	0.8529	1.1052	Translation fidelity
At4g35150	O-methyltransferase family 2 protein similar to caffeic acid O-methyltransferase, Pinus taeda, gb:U39301	0.4855	0.7486	0.8236	Fenilpropanoid metabolism, response to fungus
At5g03600	GDSL-motif lipase/hydrolase family protein low similarity to family II lipase EXL3 [Arabidopsis thaliana] GI:15054386; contains Pfam profile PF00657: GDSL-like Lipase/Acylhydrolase	0.3057	1.284	1.1004	Lipid metabolism
At5g10000	Ferredoxin family protein similar to Ferredoxin, chloroplast precursor from Arabidopsis thaliana [SP|P16972]; contains Pfam profile: PF00111 2Fe-2S iron-sulfur cluster binding domains	0.4305	0.8125	0.8671	Electron transport, tolerance to water stress
At5g22110	DNA polymerase epsilon subunit B family contains Pfam profile: PF04042 DNA polymerase epsilon subunit B	0.4779	0.9252	0.9548	Transcription
At5g25990	Expressed protein contains Pfam profile PF03267: Arabidopsis protein of unknown function, DUF266; expression supported by MPSS	0.338	0.9857	0.8289	Carbohydrate metabolism
At5g37910	Seven in absentia (SINA) family protein similar to SIAH1 protein [Brassica napus var. Napus] GI:7657876; contains Pfam profile PF03145: Seven in absentia protein family	0.3817	0.7278	0.8343	Ubiquitination
At5g38320	Expressed protein ; expression supported by MPSS	0.312	0.998	0.7281	---
At5g38810	F-box family protein predicted proteins, Arabidopsis thaliana	0.4925	0.7123	0.6849	---
At5g46090	Expressed protein contains Pfam profile PF05078: Protein of unknown function (DUF679); expression supported by MPSS	0.4617	0.8625	1.2554	---
At5g48100	Laccase family protein / diphenol oxidase family protein similar to laccase [Pinus taeda][GI:13661197]	0.4369	1.013	0.8338	Carbohydrate metabolism
At5g53520	Oligopeptide transporter OPT family protein similar to SP|P40900 Sexual differentiation process protein isp4 {Schizosaccharomyces pombe}, oligopeptide transporter Opt1p [Candida albicans] GI:2367386; contains Pfam profile PF03169: OPT oligopeptide transpo	0.4717	0.9981	0.813	---
At5g62830	F-box family protein-related similar to F-box protein family, atfbx9 (GI:20197985) [Arabidopsis thaliana]; contains TIGRFAM TIGR01640 : F-box protein interaction domain	0.4744	1.0579	0.7949	Ubiquitination
At5g62830	Mirna gene Arabidopsis thaliana mir395f stem-loop	0.4064	1.3782	0.9203	Respond to Pi and sulfate starvation
At5g46730	Glycine-rich protein	0.3734	0.4086	0.6978	Cell elongation, respond to pathogens
**Group II**					
At1g11210	Expressed protein contains similarity to cotton fiber expressed protein 1 [Gossypium hirsutum] gi|3264828|gb|AAC33276	1.0381	0.3689	0.7753	Respond to ABA, cold, oxidative stress and water deprivation
At1g64170	Cation/hydrogen exchanger, putative (CHX16) monovalent cation:proton antiporter family 2 (CPA2) member, PMID:11500563	0.7013	0.3702	1.3109	Cation transport
At2g02120	Plant defensin-fusion protein, putative (PDF2.1) plant defensin protein family member, personal communication, Bart Thomma (Bart.Thomma@agr.kuleuven.ac.be); contains a gamma-thionin family signature (PDOC00725)	0.7873	0.2353	0.7105	Defensin
At2g23030	Protein kinase, putative similar to protein kinase 3 [Glycine max] GP|310582|gb|AAB68961	1.018	0.4512	1.4254	Iron starvation, nitrate transport, respond to

					osmotic stress
At2g29310	Tropinone reductase, putative / tropine dehydrogenase, putative similar to tropinone reductase SP:P50165 from [Datura stramonium]	0.6255	0.4518	0.6836	Oxidation-reduction process
At2g38465	Expressed protein	0.8395	0.4115	0.7629	---
At2g40080	Expressed protein	1.0768	0.278	0.8084	Photoperiodism
At3g13130	Hypothetical protein	0.7882	0.4131	0.7214	---
At3g13160	Pentatricopeptide (PPR) repeat-containing protein contains Pfam profile PF01535: PPR repeat	1.0589	0.4741	0.7293	Development
At3g15500	No apical meristem (NAM) family protein (NAC3) identical to atnac3 [Arabidopsis thaliana] GI:12060424; contains Pfam PF02365: No apical meristem (NAM) domain; similar to jasmonic acid 2 GB:AAF04915 from [Lycopersicon esculentum]	1.103	0.4661	0.6747	MAPK cascade, ABA signaling, defense to fungus, salt, JA biosynthesis
At3g15630	Expressed protein	0.9647	0.4714	1.045	---
At3g48360	Speckle-type POZ protein-related contains Pfam PF00651 : BTB/POZ domain; similar to Speckle-type POZ protein (SP:O43791) [Homo sapiens]	0.9779	0.4926	1.2816	Respond to ABA and auxins, JA, SA, salt, and wounding
At4g22660	F-box family protein contains Pfam PF00646: F-box domain; similar to F-box protein family, atfbx7 (GI:20197899) [Arabidopsis thaliana]	0.9168	0.4921	0.7716	---
At5g24470	Pseudo-response regulator 5 (APRR5) identical to pseudo-response regulator 5 GI:10281006 from [Arabidopsis thaliana]	1.0605	0.4512	1.1884	Photoperiodism
**Group III**					
At1g11530	Thioredoxin family protein similar to thioredoxin H-type from Arabidopsis thaliana SP|P29448, Nicotiana tabacum SP|Q07090; contains Pfam profile: PF00085 Thioredoxin	0.811	0.8014	0.4088	Cell redox homeostasis
At1g16120	Wall-associated kinase, putative contains similarity to wall-associated kinase 4 GI:3355308 from [Arabidopsis thaliana]	0.7772	0.6796	0.4277	Protein phosphorylation
At1g16250	Kelch repeat-containing F-box family protein contains Pfam profiles PF01344: Kelch motif, PF00646: F-box domain	0.7406	0.836	0.3495	---
At1g18010	Expressed protein contains 11 transmembrane domains;	1.0171	0.9442	0.4838	Transmembrane transport
At1g23120	Major latex protein-related / MLP-related low similarity to major latex protein {Papaver somniferum}[GI:294060]; Location of EST gb|T88564 ; contains Pfam profile PF00407: Pathogenesis-related protein Bet v I family	1.0504	1.2336	0.4385	Responde to biotic stimulus
At1g36640	Expressed protein	0.9728	0.746	0.3071	---
At1g43160	AP2 domain-containing protein RAP2.6 (RAP2.6) identical to AP2 domain containing protein RAP2.6 GI:2281637 from [Arabidopsis thaliana]	0.9172	1.4339	0.3729	Celular response to heat, JA, cold, fungus, SA, and salt
At1g52325	Hypothetical protein	0.9154	0.7813	0.2853	Translation
At1g61480	S-locus protein kinase, putative similar to receptor protein kinase (IRK1) GI:836953 from [Ipomoea trifida]; contains S-locus glycoprotein family domain, Pfam:PF00954	0.9038	1.0583	0.4626	Protein phosphorylation
At1g74590	Glutathione S-transferase, putative similar to putative glutathione S-transferase GB:CAA10060 [Arabidopsis thaliana]; contains Pfam profile: PF00043 Glutathione S-transferases	1.1765	0.8633	0.4997	Toxin catabolism process
At1g77010	Pentatricopeptide (PPR) repeat-containing protein contains INTERPRO:IPR002885 PPR repeats	0.8361	0.9247	0.4554	---
At1g79720	Aspartyl protease family protein contains Pfam domain, PF00026: eukaryotic aspartyl protease	1.1039	0.9225	0.364	Proteolysis

At2g07671	H+-transporting two-sector ATPase, C subunit family protein similar to ATPase subunit 9 [Arabidopsis thaliana] GI:15215920; contains Pfam profile PF00137: ATP synthase subunit C	1.0194	1.6448	0.3983	Ion transport
At2g25950	Expressed protein	1.0998	1.305	0.454	Possible proteosome interaction
At2g36890	Myb family transcription factor (MYB38) contains Pfam profile: PF00249 myb-like DNA-binding domain	0.8841	1.4023	0.4202	Transcription
At2g38870	Protease inhibitor, putative similar to SP|P24076 Glu S.griseus protease inhibitor (BGIA) {Momordica charantia}; contains Pfam profile PF00280: Potato inhibitor I family	1.1226	0.9532	0.4252	Defense responses to fungus and wounding, proline transport
At2g41120	Expressed protein	0.9639	1.302	0.4204	---
At2g42720	F-box family protein contains F-box domain Pfam:PF00646	0.9085	1.2364	0.4508	DNA metabolism
At2g46510	Basic helix-loop-helix (bhlh) family protein contains Pfam profile: PF00010 helix-loop-helix DNA-binding domain	1.2193	1.0399	0.4966	ABA, auxin, ethylene signaling, responses to fungus and water stress
At3g03880	Expressed protein	0.7938	1.0566	0.3964	---
At3g10110	Mitochondrial import inner membrane translocase subunit Tim17/Tim22/Tim23 family protein weak similarity to TIM22 preprotein translocase [Rattus norvegicus] GI:6760457; contains Pfam profile PF02466: Mitochondrial import inner membrane translocase subunit	1.1191	0.8471	0.4854	Protein metabolism
At3g13940	Expressed protein weak similarity to DNA-directed RNA polymerase I 49 kda polypeptide (EC 2.7.7.6) (A49) (Swiss-Prot:O14086) [Schizosaccharomyces pombe]; similar to Nuclear pore complex protein Nup107 (Nucleoporin Nup107) (107 kda nucleoporin) (p105) (Swi	1.6065	1.4468	0.4626	Transcription
At3g28610	AAA-type ATPase family protein contains Pfam profile: ATPase family PF00004	0.6392	0.9056	0.3697	Import to nucleous
At3g60620	Phosphatidate cytidylyltransferase family protein contains Pfam profile: PF01148 phosphatidate cytidylyltransferase	1.1392	1.2467	0.4604	Phospholipid metabolism
At4g15730	Expressed protein	1.1551	0.6393	0.4773	Ion binding
At4g19170	9-cis-epoxycarotenoid dioxygenase, putative / neoxanthin cleavage enzyme, putative / carotenoid cleavage dioxygenase, putative similar to 9-cis-epoxycarotenoid dioxygenase [Phaseolus vulgaris][GI:6715257]; neoxanthin cleavage enzyme, Lycopersicon esculent	1.0226	1.1053	0.3587	Anthocyanin biosynthesis
At4g36580	AAA-type ATPase family protein contains Pfam domain, PF00004: ATPase, AAA family (ATPases associated with diverse cellular activities)	1.1735	0.8415	0.4563	Development
At5g37730	Expressed protein	1.0504	0.6247	0.3514	---
At5g46380	Hypothetical protein	0.8741	1.0426	0.3808	---
At5g54220	Hypothetical protein	0.8204	1.0874	0.4584	Cell fate specification
