# Supplementary material for: An improved, low-cost, hydroponic system for growing Arabidopsis and other plant species under aseptic conditions
Source: BMC Plant Biol. 2014 Mar 21;14:69. doi: 10.1186/1471-2229-14-69 (PMC3999955; doi:10.1186/1471-2229-14-69)
Supplement: Additional file 4 — qRT-PCR primers used in this study. [file 1471-2229-14-69-S4.pdf]

**Additional file 4.** qRT-PCR primers used in this study

| Locus | Oligonucleotide name | Oligonucleotide sequence (5’-3’) |
|---|---|---|
| AT4G28610 | PHR1FW | aaaccaacccggcgattca |
| | PHR1RW | agcggtgtcaacttcctttctgg |
| AT3G25710 | BHLH32FW | gttcgggtcaaacttgttctctg |
| | BHLH32RW | tatcgcttaggccttcgcct |
| AT2G33770 | PHO2FW | ctcatgagccaccaatggtacat |
| | PHO2RW | tcggcccaactgcttatcata |
| At5g23630 | Pdr2FW | ggagcactgaagcaggcccatgtt |
| | Pdr2RW | ttgaacatctgaagagtcgtcacaagt |
| AT5G13080 | WRKY75FW | atgggtcgttgtatgctcct |
| | WRKY75RW | cacctatagtaactcctaggg |
| AT1G23010 | LPR1FW | tcagggactacagaggtatggga |
| | LPR1RW | accttaagcggcctcatcat |
| AT5G20150 | SPX1FW | tccctgctaacgaaactgagtct |
| | SPX1REV | ggcggcaatgaaaacacact |
| AT3G05630 | PLDZ2FW | cgaatgaacatatacgctcaagagctg |
| | PLDZ2REV | agcagtgaagtagaactcgacttgagg |
| AT3G18780 | ACT2FW | gaattgtctcgttgtcctcctcac |
| | ACT2REV | tgcaaatccagccttcaccatac |
| AT5G55840 | PPRFW | tgcgactacatacaaagctctcatcag |
| | PPRREV | tgttactttgcaaggccttcagcttctc |
| AT3G53090 | UBHECTFW | cgtgctacaatactcttaagcttccaac |
| | UBHECTREV | agaggtctctacgagagttcaaatcct |
